# Alpinumisoflavone Impairs Mitochondrial Respiration via Oxidative Stress and MAPK/PI3K Regulation in Hepatocellular Carcinoma Cells

**DOI:** 10.3390/antiox11101929

**Published:** 2022-09-28

**Authors:** Hyewon Jang, Jiyeon Ham, Jisoo Song, Gwonhwa Song, Whasun Lim

**Affiliations:** 1 Department of Biological Sciences, College of Science, Sungkyunkwan University, Suwon 16419, Korea; 2 Institute of Animal Molecular Biotechnology, Department of Biotechnology, College of Life Sciences and Biotechnology, Korea University, Seoul 02841, Korea

**Keywords:** alpinumisoflavone, liver cancer, oxidative stress, OXPHOS, calcium homeostasis

## Abstract

Alpinumisoflavone is a natural prenylated isoflavonoid extracted from the raw fruit of *Cudrania tricuspidata*. Several studies have reported the beneficial characteristics of alpinumisoflavone, such as its antioxidant, anti-inflammation, anti-bacterial, osteoprotective, and neuroprotective effects. Alpinumisoflavone also has anti-cancer effects on thyroid, renal, and ovarian cancers, but its therapeutic effects on hepatocellular carcinoma (HCC) have not yet been demonstrated. We investigated the anti-cancer effects of alpinumisoflavone on HCC using human liver cancer cell lines, Hep3B and Huh7. Our results confirmed that alpinumisoflavone inhibited viability and regulated the MAPK/PI3K pathway in Hep3B and Huh7 cells. We also verified that alpinumisoflavone can depolarize the mitochondrial membrane potential and suppress the mitochondrial respiration in HCC cells. Moreover, we confirmed the dysregulation of the mitochondrial complexes I, III, and V involving mitochondrial oxidative phosphorylation at the mRNA level and the accumulation of calcium ions in the mitochondrial matrix. Lastly, we demonstrated that alpinumisoflavone induced mitochondria-mediated apoptosis via regulation of the Bcl-xL and BAK proteins. This study elucidates the anti-cancer effects of alpinumisoflavone on HCC.

## 1. Introduction

Hepatocellular carcinoma (HCC) is one of the most diagnosed malignant tumors, currently designated as the cancer with the third highest mortality [1]. Causes of HCC include chronic viral infection, non-alcoholic fatty liver disease, inflammation, and exposure to toxic compounds, which could induce oxidative DNA damage [2]. The first-choice treatment for early liver cancer patients is surgery, with a post-surgery 5-year survival rate exceeding 70%; however, the recurrence rate of HCC is quite high even after hepatic resection due to its high invasiveness and metastatic capacity [3]. Additionally, sorafenib, the most common anti-cancer drug for HCC treatment, has nonnegligible side effects, including fatigue, weight loss, rash, hair loss, erythema, diarrhea, anorexia, nausea, and abdominal pain [4]; thus, new alternatives or aiding agents are needed to overcome resistance and diminish these side effects [4].

Since natural products and their derivatives have a great structural and biological diversity, lower toxic side effects, and wide source availability, their importance in the development of new anti-cancer drugs and lead drug compounds has increased in recent years [5,6,7,8]. Isoflavones, such as formononetin, have estrogen-like activity because of their structural similarity to estrogen. [9]. Moreover, isoflavones genistein and daidzein suppressed cell cycle progression and inhibited cell viability even in sorafenib-resistant HCC cells, hinting at their potential as therapeutic aids in tumor treatment [10,11].

Alpinumisoflavone, a pyran isoflavone, is one of the major bioactive compounds extracted from the raw fruit of *Cudrania tricuspidata* [12], with reported antioxidant, anti-inflammation, estrogenic, and neuroprotective effects [13]. Alpinumisoflavone could further prevent the progression of osteoporosis and improve the cholesterol profile in blood [14], as well as enhance the sensitivity to apoptosis in radioresistant esophageal squamous cell carcinoma [15]. In addition, several studies have proven that alpinumisoflavone could induce apoptosis in kidney cancer via miRNA regulation and in human ovarian cancer via mitochondrial dysfunction [16,17]. However, few studies have shown the anti-cancer effects of alpinumisoflavone as a liver cancer treatment.

Therefore, we conducted experiments to demonstrate the effects and mechanisms of alpinumisoflavone on HCC cells using cell lines Hep3B and Huh7; we focused on investigating (1) cellular viability, (2) intracellular signaling pathways with or without pharmacological inhibitors, (3) oxidative stress and mitochondrial energy metabolism, (4) mitochondrial calcium homeostasis, and (5) mitochondrial-mediated apoptosis. Our results suggest that alpinumisoflavone could be utilized as a potential therapeutic agent to treat HCC.

## 2. Materials and Methods

### 2.1. Reagents and Chemicals

Alpinumisoflavone (Cat No. CFN98440) was purchased from ChemFaces (Wuhan, China) and dissolved in dimethyl-sulfoxide (DMSO). The utilized antibodies against the target proteins are listed in Table 1. Sorafenib (Cat No. SML2653) was purchased from Sigma-Aldrich and was dissolved in DMSO.

### 2.2. Cell Culture

The hepatocarcinoma-derived cell lines Hep3B and Huh7 were obtained from the Korea Cell Line Bank (KCLB; Seoul, Korea) and maintained according to the suggested guidelines (https://cellbank.snu.ac.kr, accessed on 18 July 2022). Briefly, the cells were maintained in a 100-mm cell culture dish until 70% confluence was reached; afterward, the cells were cultured in a 100- or 60-mm cell culture dish or a 96-well plate depending on the subsequent assay. After cell confluency reached 70%, various concentrations of alpinumisoflavone (0, 5, 10, 20, and 50 µM), with or without inhibitors, were added to serum-free medium for 48 h [18]. 

### 2.3. Detection of Cell Viability Based on Metabolic Activity

The Hep3B and Huh7 cells were cultured in a 96-well culture dish and treated with various concentrations of alpinumisoflavone (0, 5, 10, 20, and 50 µM), with or without 20 µM U0126 (extracellular signal-regulated protein kinase (ERK)1/2 inhibitor) and SP600125 (c-Jun N-terminal kinase (JNK) inhibitor), for 48 h, following the indications detailed in a previous study [19]. The absorbance at 560 and 650 nm (yellow to purple) was detected using an ELISA plate reader, using the BioTek Gen5 software.

### 2.4. Hanging-Drop Assay for Spheroid Formation 

Hep3B and Huh7 cell suspensions were prepared at a concentration of 12,000 cells per 25 μL drop in serum-containing medium. Alpinumisoflavone (50 µM) or DMSO-treated cell suspensions were seeded to the lids of culture dishes and maintained for 72 h for imaging purposes. The ImageJ software was used for analysis of the spheroid surface [20].

### 2.5. Detection of a Proliferation Marker via Immunofluorescence

The expression of proliferative cell nuclear antigen (PCNA) (Cat. No. sc-56, Santa Cruz Biotechnology, Santa Cruz, CA, USA) was quantified by immunofluorescence. The cells were seeded in a confocal dish and treated with either alpinumisoflavone (50 µM) or DMSO (vehicle). After methanol fixation, PCNA and a goat anti-mouse IgG Alexa 488 antibody were serially incubated with the cells to visualize the green fluorescence. Lastly, after several washing steps, the nuclei were labeled with 5 µg/mL 4′, 6-diamidino-2-phenylindole (DAPI). The images were acquired using a confocal microscope (LSM710, Carl Zeiss, Oberkochen, Germany) [20,21].

### 2.6. Western Blotting

The proteins were extracted by whole-cell lysis after treatment for 30 min, with or without treatment with pharmacological inhibitors for 2 h prior to alpinumisoflavone exposure. Denatured proteins were separated via sodium dodecyl sulfate-polyacrylamide gel electrophoresis (SDS–PAGE) and transferred to nitrocellulose membranes. Immunoblots were captured using the Image Lab software. Each total protein and alpha-tubulin (TUBA) were utilized as a loading control for normalization [17].

### 2.7. JC-1 Staining Assay

Changes in the JC-1 aggregate to the monomer relative ratio were studied using a mitochondrial staining kit (Cat. No: CS0390, Sigma-Aldrich, St. Louis, MO, USA) to detect the mitochondrial membrane potential (MMP). Hep3B and Huh7 cells (6 × 10^5^ cells) were seeded into 60-mm cell culture plates and treated with alpinumisoflavone in a dose-dependent manner (0, 5, 10, 20, and 50 µM) for 48 h at 37 °C in a CO_2_ incubator. Alpinumisoflavone-treated cells were collected and stained with JC-1 dye at a concentration of 5 µg/mL, following the experimental protocol detailed in a previous study. Analysis was performed using a FACS Calibur instrument (BD Biosciences, Flanklin Lakes, NJ, USA) and the FlowJo software [22].

### 2.8. Mito-Stress Assay Using the Seahorse XF-24 Analyzer

Hep3B and Huh7 cells (3 × 10^4^ cells/well) were seeded into a 24-well Seahorse XF plate to measure the oxygen consumption rate under alpinumisoflavone (50 µM) or DMSO treatment for 24 h. The Seahorse XF analyzer (Agilent Technologies, Santa Clara, CA, USA) was operated under the following conditions: Oligomycin (1 µM), FCCP (1 µM), and Rotenone/Antimycin A (0.5 µM) serial treatment [23].

### 2.9. Real-Time Polymerase Chain Reaction (RT-PCR) Analysis

Total RNA was extracted using the Transzol-up reagent (Cat No. #ET111-01, TransGen Biotech Co., Beijing, China) after treating the Hep3B and Huh7 cells with alpinumisoflavone for 48 h, and complementary DNA was synthesized for mRNA quantitative analysis. The *GAPDH* gene was used for normalization. The experiment was performed following the indications described in a previous study [18]. The primers for the target genes are listed in Table 2.

### 2.10. Rhod-2 Staining Assay

The Hep3B and Huh7 cells were seeded into 60-mm cell culture dishes and treated as described above. Alpinumisoflavone-treated cells were collected and stained with Rhod-2 dye, following the experimental protocol described in a previous study [21]. Analysis was performed using a FACS Calibur instrument (BD Biosciences) and the FlowJo software.

### 2.11. Measurement of Apoptotic Cell Population

The Hep3B and Huh7 cells were seeded into 60-mm cell culture dishes and treated as described above. Apoptosis of Hep3B and Huh7 cells was examined using a FITC Annexin V apoptosis detection kit I (BD Biosciences), following the experimental protocol described in a previous study [22]. Analysis was performed using a FACS Calibur instrument (BD Biosciences) and the FlowJo software.

### 2.12. Statistical Analysis

The mean and standard deviation variation of the in vitro data in this study were calculated from three independent experiments, and the data were analyzed using an ANOVA test with the PROC general linear model (PROC-GLM, SAS Institute, Cary, NC, USA). Significant differences between the vehicle and alpinumisoflavone- or inhibitor-treated groups were indicated with an asterisk or lowercase letters according to its probability value (* *p* < 0.05, ** *p* < 0.01, *** *p* < 0.001).

## 3. Results

### 3.1. Alpinumisoflavone Suppresses the Viability of Huh7 and Hep3B Cells

**Figure 1:**
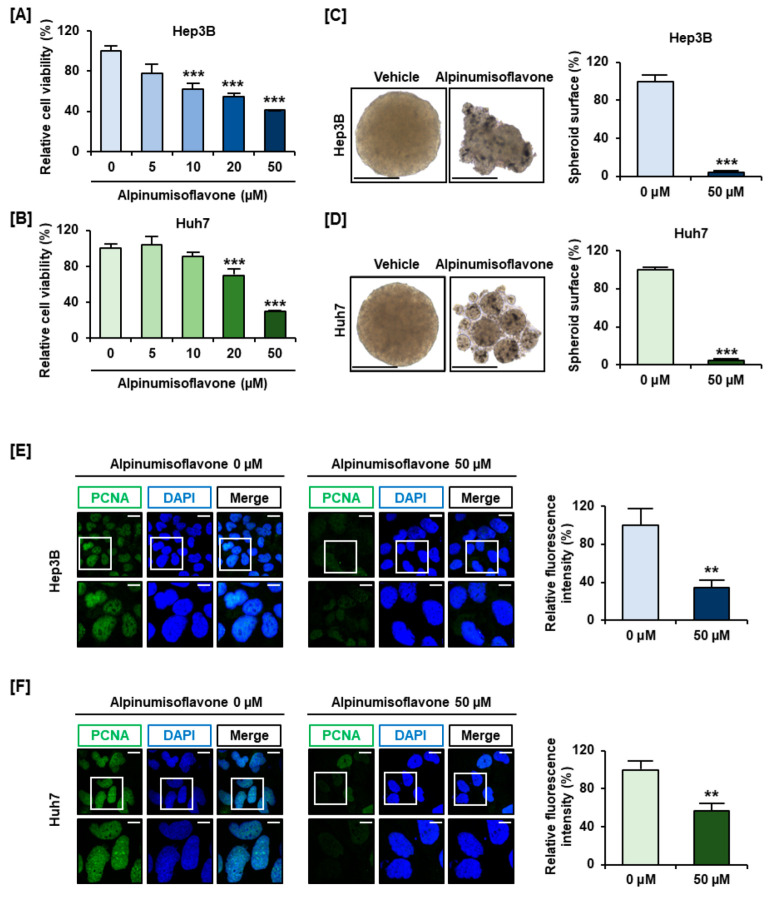
The viability evaluation in HCC cells in response to alpinumisoflavone treatment. (**A**,**B**) Hep3B and Huh7 cell proliferation in response to treatment with alpinumisoflavone (0, 5, 10, 20, and 50 µM). (**C**,**D**) Changes in 3D spheroid formation in 50 µM alpinumisoflavone-treated Hep3B and Huh7 cells. The corresponding ratio values are expressed as the average value of triplicate data. (**E**,**F**) PCNA immunofluorescence, indicated as green fluorescence. The nuclei were co-stained with DAPI. Scale bars indicate 20 μm in the upper image and 40 μm in the lower image. Significant differences between the control and alpinumisoflavone-treated cells are indicated with asterisks (** *p* < 0.01 and *** *p* < 0.001).

Cell viability was measured after treatment with different concentrations of alpinumisoflavone (0, 5, 10, 20, and 50 µM) in Hep3B and Huh7 cells (Figure 1A,B), and we observed that 50 µM alpinumisoflavone reduced the viability of Hep3B and Huh7 cells by up to 41% and 29%, respectively (*p* < 0.001). Moreover, 50 µM alpinumisoflavone inhibited cell spheroid formation and critically suppressed its relative surfaces under 5% in both Hep3B and Huh7 cells (*p* < 0.001) compared to vehicle cells (Figure 1C,D). We also confirmed the expression of proliferative marker PCNA as green fluorescence in both the cell lines, and it was significantly decreased to approximately 34% in Hep3B and 57% in Huh7 cells (*p* < 0.01) in 50 µM alpinumisoflavone-treated cells (Figure 1E,F), thus confirming that alpinumisoflavone reduced the viability and proliferation of HCC cells. 

### 3.2. Alpinumisoflavone Regulates the MAPK/PI3K Pathways in HCC Cells

**Figure 2:**
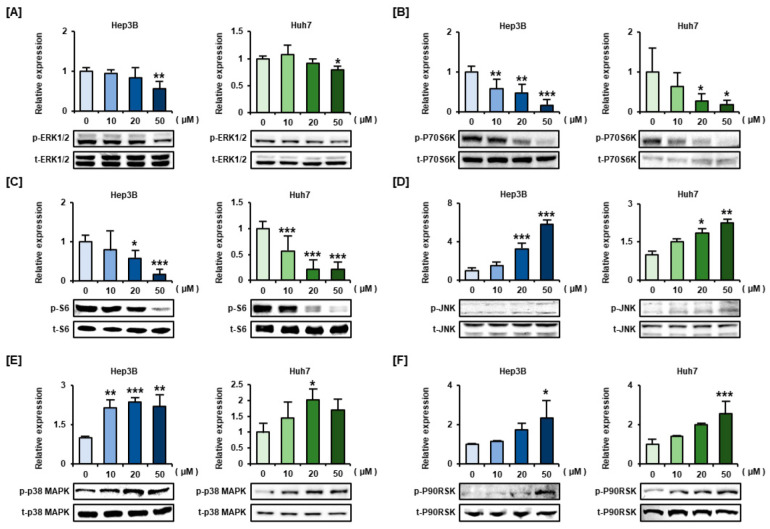
Modulation of the PI3K and MAPK signaling pathways in alpinumisoflavone-treated Hep3B and Huh7 cells. (**A**–**F**) Phosphorylation of ERK1/2 (**A**), P70S6K (**B**), S6 (**C**), JNK (**D**), p38 MAPK (**E**), and P90RSK (**F**) in response to a 30 min exposure to alpinumisoflavone (0, 10, 20 and 50 µM). Phosphorylated proteins were normalized by the amount of each total protein. Significant differences between the control and alpinumisoflavone-treated cells are indicated with asterisks (* *p* < 0.05, ** *p* < 0.01, and *** *p* < 0.001).

To unveil the intracellular mechanisms related to the viability and proliferation of HCC cells, western blotting was performed after a 30 min exposure of Huh7 and Hep3B cells to alpinumisoflavone (0, 10, 20, and 50 µM) (Figure 2). The relative phosphorylation of ERK1/2 in alpinumisoflavone-treated Hep3B and Huh7 cells was slightly decreased to 41% and 21%, respectively, compared to that in control cells (Figure 2A). In the case of the phosphor-P70S6K and S6 proteins downstream of ERK1/2, the relative phosphorylation was significantly reduced under 22% by alpinumisoflavone in a dose-dependent manner in Hep3B and Huh7 cells (Figure 2B,C). In contrast, the phosphorylation levels of JNK, p38, and P90RSK increased proportionally when the concentration of alpinumisoflavone was increased in both the HCC cell lines (Figure 2D–F).

### 3.3. Interactions in the MAPK Signaling Pathway Are Altered by Alpinumisoflavone

**Figure 3:**
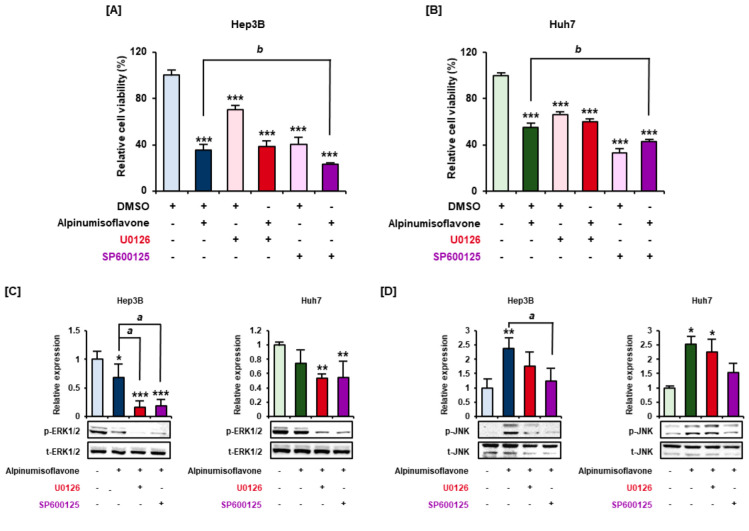
Cell viability and MAPK signaling changes in response to treatment with alpinumisoflavone in the presence or absence of pharmacological inhibitors. (**A**,**B**) Cell viability was analyzed for 48 h after pre-treatment with U0126 (ERK1/2 inhibitor) and SP600125 (JNK inhibitor) and with or without treatment with 50 µM alpinumisoflavone. (**C**,**D**) The cells were treated with pharmacological inhibitors for 2 h prior to alpinumisoflavone treatment for 30 min, and the phosphorylation changes in ERK1/2 (**C**) and JNK (**D**) were observed and represented graphically. Significant differences between the control and alpinumisoflavone-treated cells are indicated with asterisks (* *p* < 0.05, ** *p* < 0.01, and *** *p* < 0.001). Lowercase letters indicate significant differences (^a^ *p* < 0.05 and ^b^ *p* < 0.01).

To determine the interactions between the MAPK pathway and cell viability in alpinumisoflavone-treated cells, experiments were conducted using 50 µM alpinumisoflavone and 20 µM MAPK inhibitors. Individual treatment with the ERK1/2 inhibitor U0126 and the JNK MAPK inhibitor SP600125 significantly decreased the viability of Hep3B and Huh7 cells (Figure 3A,B). Co-treatment with alpinumisoflavone and U0126 did not reveal any synergetic effects on cell viability, but co-treatment with SP600125 suppressed it more than individual treatment with alpinumisoflavone in both Hep3B and Huh7 cells (Figure 3A,B). Furthermore, we performed western blot analyses to determine the influence of alpinumisoflavone on the regulation mechanism of the intracellular signaling pathway by treating the cells with 20 µM pharmacological inhibitors for 2 h prior to treatment with alpinumisoflavone for 30 min. Pre-treatment with U0126 and SP600125 followed by treatment with alpinumisoflavone critically decreased the expression levels of phosphor-ERK1/2 by 20% in Hep3B and 55% in Huh7 cells, compared with those in vehicle cells (Figure 3C). SP600125 did not only affect JNK but also downregulated phosphor-ERK1/2 more than that in alpinumisoflavone-treated Hep3B cells. In contrast, no significant changes between alpinumisoflavone-treated and alpinumisoflavone and SP600125 co-treated Huh7 cells were noted. Additionally, p-JNK expression was significantly suppressed by SP600125 compared to that in alpinumisoflavone-treated Hep3B cells (Figure 3D), but it was alleviated by SP600125 pre-treatment in Huh7 cells (Figure 3D). Pre-treatment with U0126 and alpinumisoflavone did not critically change the p-JNK expression in any of the two cell lines (Figure 3D). These results indicated that alpinumisoflavone inhibits the viability of HCC cells by regulating MAPK signals.

### 3.4. Alterations in the MMP Caused by Alpinumisoflavone Elicited Energy Metabolism Disruption in HCC Cells

**Figure 4:**
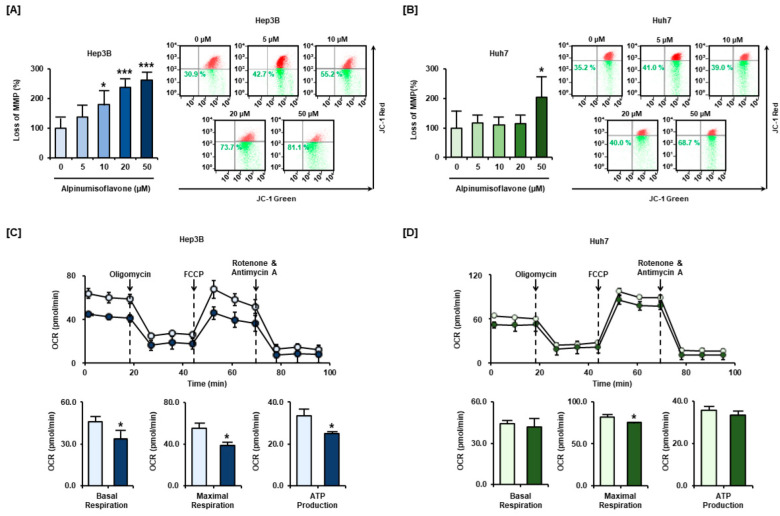
Alpinumisoflavone treatment induced loss of MMP and changes in mitochondrial respiration. (**A**,**B**) MMP depolarization was evaluated via JC-1 dye accumulation. (**C**,**D**) A Seahorse XF analyzer was used to evaluate oxygen consumption, basal and maximal respiration, and ATP production after alpinumisoflavone treatment, and the observed changes can be confirmed in the bar graphs. Significant differences between the control and alpinumisoflavone-treated cells are indicated with asterisks (* *p* < 0.05 and *** *p* < 0.001).

The mitochondrial function was changed in a monolayer culture of HCC cells treated with alpinumisoflavone (0, 5, 10, 20, and 50 µM). In Hep3B cells, the loss of MMP, quantified as the JC-1 green dye proportion, was increased to 138%, 178%, 238%, and 262% upon treatment with 5, 10, 20, and 50 µM alpinumisoflavone, respectively, compared to vehicle cells (Figure 4A). Similarly, the proportion of JC-1 green dye in Huh7 cells was increased to 116%, 110%, 113%, and 203% upon treatment with 5, 10, 20, and 50 µM alpinumisoflavone, respectively, compared to that in vehicle cells (Figure 4B). After we confirmed the depolarization of the mitochondrial membrane, we further examined the mitochondrial oxidative phosphorylation system (OXPHOS) using a Seahorse XF analyzer, and we observed that, in Hep3B cells, alpinumisoflavone induced notable changes in basal and maximal respiration and in ATP production, which decreased by 28%, 31%, and 26%, respectively (Figure 4C). However, there were only slight changes in the respiration capacity of alpinumisoflavone-treated Huh7 cells (Figure 4D). Collectively, we confirmed that alpinumisoflavone induced mitochondrial dysfunction in HCC cell lines.

### 3.5. Alpinumisoflavone Regulated Mitochondrial Respiratory Chain Complex I, III, and V Related Genes in Human HCC Cells

**Figure 5:**
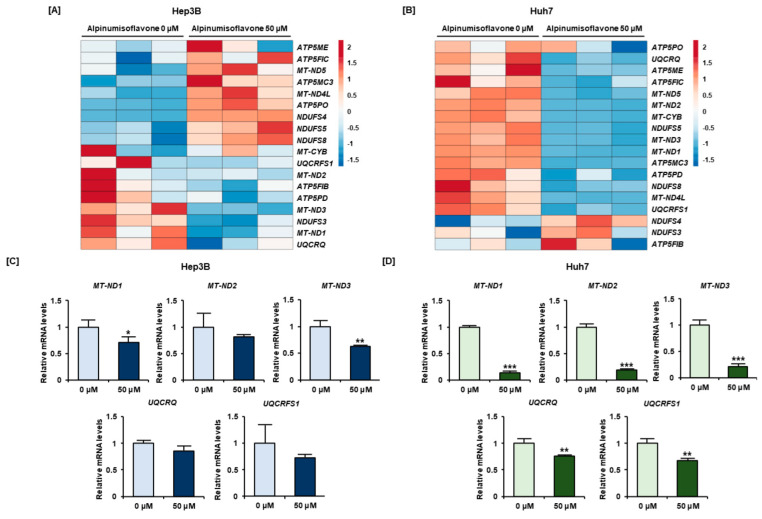
Differential expression patterns of mitochondrial respiratory complex-related genes in Hep3B and Huh7 cells. (**A**,**B**) Heat maps of the mitochondrial respiratory complex-related genes in Hep3B and Huh7 cells, obtained with the ClustVis tool and based on RT- PCR data. The color classification box in the upper right corner of the heat map symbolizes a comparative statement value, with red indicating upregulation and blue indicating downregulation. The name of each gene is specified on the right side of the heat map plot. (**C**,**D**) Quantitative analysis of the mRNA levels of *MT-ND1, MT-ND2, MT-ND3, UQCRQ,* and *UQCRFS1,* normalized to that of the housekeeping gene *GAPDH*. Significant differences between the control and alpinumisoflavone-treated cells are indicated with asterisks (* *p* < 0.05, ** *p* < 0.01 and *** *p* < 0.001).

We subsequently studied the alpinumisoflavone-induced alterations in the expression of mitochondrial complex-related genes in HCC cells via RT-PCR, and verified that alpinumisoflavone regulated the mRNA expression of mitochondrial respiratory chain complex-related genes (Figure 5A,B). In both Hep3B and Huh7 cells, the *mitochondrially encoded NADH: ubiquinone oxidoreductase core subunit (MT-ND)* and *ubiquinol-cytochrome c reductase* (*UQCR*) gene families, which include the mitochondrial complexes I and III, respectively, were downregulated by alpinumisoflavone treatment (Figure 5A,B). The mRNA expression of ATP synthase-related genes, encoded by mitochondrial complex V, was upregulated in Hep3B cells, whereas it was downregulated in Huh7 cells (Figure 5A,B). Alpinumisoflavone also downregulated mitochondrial complex I genes *MT-ND1*, *MT-ND2*, and *MT-ND3* by 30%, 19%, and 37% in Hep3B cells, and by 87%, 81%, and 79% in Huh7 cells, respectively, compared to that in vehicle cells (Figure 5C,D). Moreover, alpinumisoflavone downregulated mitochondrial complex III genes *UQCRQ* and *UQCRFS1* by 15% and 82% in Hep3B cells, and by 24% and 34% in Huh7 cells, respectively, compared to that in vehicle cells (Figure 5C,D). In summary, alpinumisoflavone downregulated mitochondrial complex I and III genes.

### 3.6. Alpinumisoflavone Disrupts the Mitochondrial Calcium Homeostasis in HCC Cells

**Figure 6:**
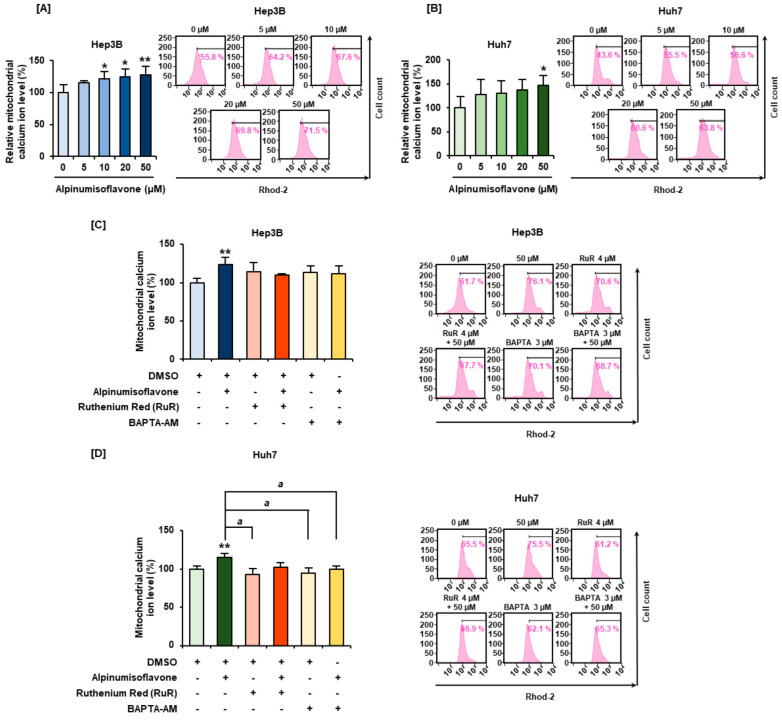
Effects of alpinumisoflavone treatment on mitochondrial matrix calcium homeostasis in Hep3B and Huh7 cells. (**A**,**B**) Relative mitochondrial calcium levels in Hep3B and Huh7 cells were measured using Rhod-2 dye. (**C**,**D**) Hep3B and Huh7 cells were co-treated with intercellular calcium chelator BAPTA-AM (3 µM) or mitochondrial calcium chelator ruthenium red (4 µM) and alpinumisoflavone (50 µM) for 48 h. Significant differences between the control and alpinumisoflavone-treated cells are indicated with asterisks (* *p* < 0.05 and ** *p* < 0.01). A lowercase letter indicates significant differences (^a^ *p* < 0.05).

To establish the change in calcium concentration in the mitochondria, HCC cells were treated with 0, 5, 10, 20, and 50 µM alpinumisoflavone, and Rhod-2 fluorescence was subsequently observed. Calcium levels in the mitochondria in Hep3B cells increased to 125% and 128% upon treatment with 20 and 50 µM alpinumisoflavone, respectively, compared to that in vehicle cells (Figure 6A). In Huh7 cells, mitochondrial calcium levels increased up to 146% upon treatment with 50 µM alpinumisoflavone, compared with that in vehicle cells (Figure 6B). The chelation of calcium ions in the mitochondria using Ruthenium Red and BAPTA-AM restored the alpinumisoflavone-induced intramitochondrial calcium accumulation to normal levels in Hep3B and Huh7 cells (Figure 6C,D).

### 3.7. Alpinumisoflavone Induces Mitochondrial Apoptosis in Hep3B and Huh7 Cells

**Figure 7:**
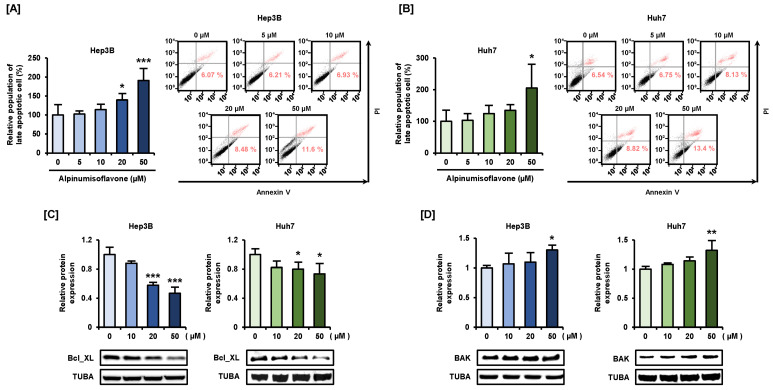
Alpinumisoflavone can induce the apoptotic cell death of Hep3B and Huh7 cells. (**A**,**B**) Late apoptotic cell death of alpinumisoflavone-treated Hep3B and Huh7 cells was analyzed using Annexin V and PI. The relative cell population in the upper right panel (late apoptotic cells) was transformed into a percentage ratio graph. (**C**,**D**) Immunoblots and relative expression of Bcl-xL (**C**) and BAK (**D**); each protein was normalized using TUBA as total protein. Significant differences between the control and alpinumisoflavone-treated cells are indicated with asterisks (* *p* < 0.05, ** *p* < 0.01, and *** *p* < 0.001).

Lastly, we aimed to demonstrate whether alpinumisoflavone could induce cell death in HCC cells and, if so, which kind of cell death was induced. We observed that the number of late apoptotic Hep3B and Huh7 cells increased proportionally to the concentration of alpinumisoflavone. The late apoptotic cell rates of Hep3B and Huh7 cells were increased to 190% (*p* < 0.001) and 205% (*p* < 0.05) by alpinumisoflavone, respectively, compared with that of vehicle cells (Figure 7A,B). Based on our results regarding the effects of alpinumisoflavone on mitochondrial dysfunction, we further confirmed the expression of the Bcl-xL and BAK proteins, which mediate mitochondrial apoptosis, via western blot analysis. Alpinumisoflavone-treated Huh7 and Hep3B cells showed a remarkable decrease in the expression levels of anti-apoptotic protein Bcl-xL phosphorylation (Figure 7C), while the expression levels of pro-apoptotic protein BAK increased in proportion to that of alpinumisoflavone concentration up to a 1.3-fold change in both the HCC cell lines (Figure 7D). This finding confirmed that the apoptosis of HCC cells was induced by alpinumisoflavone and mediated by mitochondria.

### 3.8. Combined Effect of Alpinumisoflavone and Sorafenib in Hep3B and Huh7 Cells

**Figure 8:**
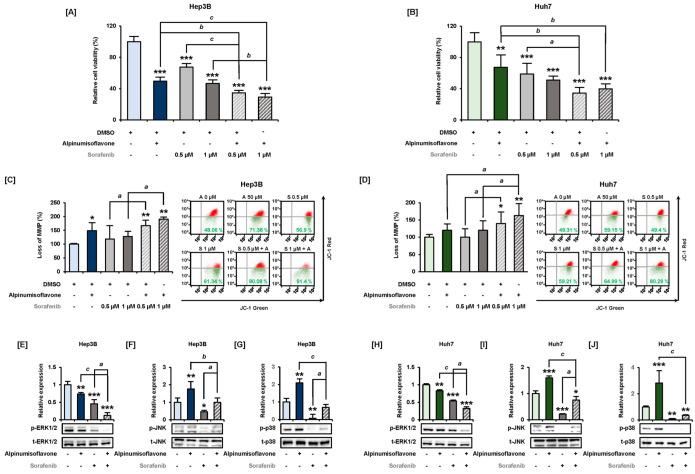
Combined effect of alpinumisoflavone and sorafenib on Hep3B and Huh7 cells. (**A**,**B**) Hep3B and Huh7 cell viability in response to treatment with alpinumisoflavone (0–50 µM) and sorafenib (0.5–1.0 µM). (**C**,**D**) Evaluation of the depolarization of mitochondrial membrane via JC-1 dye accumulation under alpinumisoflavone (50 µM) and sorafenib (0.5 and 1 µM) treatment for 48 h. (**E**–**J**) Immunoblots and relative expression of phosphor-ERK1/2 (**E**,**H**), phosphor-JNK (**F**,**I**), and phosphor-p38 MAPK (**G**,**J**) in Hep3B and Huh7 cells, in response to alpinumisoflavone (50 µM) with or without sorafenib (1 µM). Each protein was normalized using each total protein. Significant differences between the control and alpinumisoflavone-treated cells are indicated with asterisks (* *p* < 0.05, ** *p* < 0.01, and *** *p* < 0.001). Significant differences between single-drug treatment and co-treatment groups are indicated with lowercase letters (* *a* < 0.05, ** *b* < 0.01, and *** *c* < 0.001).

To confirm the therapeutic effect and mechanisms of alpinumisoflavone in combination with sorafenib, we performed a viability assay. Single-drug treatments with sorafenib and alpinumisoflavone suppressed the viability of Hep3B to 49% and 67%, respectively, whereas co-treatment with sorafenib (0.5 μM) and alpinumisoflavone (50 μM) significantly suppressed the viability to 34% (Figure 8A). Single-drug treatments with alpinumisoflavone and sorafenib suppressed the viability of Huh7 cells to 67% and 59%, respectively, whereas co-treatment with alpinumisoflavone and sorafenib (0.5 μM) significantly suppressed the viability to 34% (Figure 8B). Additionally, co-treatment with sorafenib (1 μM) and alpinumisoflavone could reduce the viability of Hep3B and Huh7 cells to 29% and 40%, respectively (Figure 8A,B). Furthermore, co-treatment with alpinumisoflavone (50 μM) and sorafenib (0.5 and 1 μM) accelerated the depolarization of mitochondrial membrane by 190% and 163% as compared to control in Hep3B and Huh7 cells, respectively (Figure 8C,D). Lastly, we co-treated cells with alpinumisoflavone (50 μM) and sorafenib (1 μM) for 30 min and analyzed the MAPK signal transduction changes via western blot analysis (Figure 8E–J). The relative phosphorylation of ERK1/2 was significantly reduced by single-drug treatment with sorafenib and was further decreased to under 0.12 and 0.32 foldchanges in Hep3B and Huh7 cells, respectively, with co-treatment (Figure 8E,H). Single-drug treatment with sorafenib dramatically suppressed the phosphorylation of JNK and p38 MAPK, which tended to be restored to normal levels when administered in combination with alpinumisoflavone in both Hep3B and Huh7 cells (Figure 8F,G,I,J). 

## 4. Discussion

Alpinumisoflavone reportedly has many beneficial physiological characteristics and therapeutic effects on diseases, including cancer. However, its anti-cancer mechanisms on HCC had not been studied in depth; thus, we conducted several experiments in Hep3B and Huh7 cells to elucidate the anti-cancer effects of alpinumisoflavone. The Hep3B cell line originates from HCC patients infected with the hepatitis B virus, while Huh7 cells are derived from male hepatoma tissue [24,25]. Hep3B cells are p53 null type cells, whereas Huh7 cells have a mutation on p53 that produces a different response to radiation [26]. In this study, we have demonstrated the anti-cancer effects of alpinumisoflavone and its mechanisms, with a focus on mitochondrial function, using Hep3B and Huh7 cell lines. First, we demonstrated the anti-proliferative effects of alpinumisoflavone on Hep3B and Huh7 cells not only in adherent cultures, but also in 3D spheroids, mimicking the in vivo conditions. We also confirmed the decrease in the levels of typical proliferation marker PCNA via immunofluorescence, suggesting a possible use of alpinumisoflavone as a therapeutic agent. 

Since MAPK and PI3K signaling is closely related to cell survival and proliferation, signaling modulation patterns were investigated. The well-known PI3K and ERK signaling pathways are involved in cellular viability, proliferation, and migration in response to various extracellular stimuli, such as growth factors [27,28,29]. Furthermore, approximately half of the HCC patients present overactivated ERK cascade signals [30], and infection with the hepatitis B virus can especially activate the ERK pathway [31]. Therefore, targeting MAPK to inactivate the kinase activity with drugs such as sorafenib is a traditional therapeutic strategy [32], but a combination therapy has been recently applied to overcome resistance and reduce its side effects. Co-treatment with alpinumisoflavone and sorafenib effectively suppressed the proliferation of HCC cells (Figure 8). Alpinumisoflavone has anti-angiogenic potential as it inhibits vascular endothelial growth factor receptor-2 (VEGFR2) [33], and VEGFR2-targeted co-treatment with ramucirumab and sorafenib is already proven to be effective in improving the overall survival of HCC patients, which implies the potential of alpinumisoflavone as a therapeutic aid [34]. Accordingly, downregulation of the ERK1/2 and PI3K signaling cascades by alpinumisoflavone might reveal clinical significance. The JNK and P38 MAPK proteins were activated under starving conditions and caused cellular apoptosis in HCC cells, although ERK1/2 phosphorylation did not change significantly in Huh7 cells [35]. We also unveiled the interactions between alpinumisoflavone and the MAPK pathway using inhibitors. Our results implied an interaction between ERK1/2 and JNK MAPK, as co-treatment with alpinumisoflavone and the JNK MAPK inhibitor SP600125 suppressed the viability of HCC cells more than treatment with alpinumisoflavone alone. The JNK inhibitor, SP600125, does not exclusively work on JNK but suppresses the activity of ERK1/2 as well. Simply blocking the ERK1/2 MAPK pathway could be critical to the viability of Hep3B and Huh7 cells, which can be inferred from our results (Figure 3A,B). Therefore, ERK1/2 is presumed to be inhibited by SP600125 via alternative pathways, resulting in a significant decrease in the viability of Hep3B and Huh7 cells. Furthermore, sorafenib is known to suppress the activity of JNK and p38 MAPK in Hep3B and Huh7 cells in a time- and dose-dependent manner [36]. In our study, co-treatment with alpinumisoflavone and sorafenib restored the activity of JNK and p38 MAPK but critically suppressed the phosphorylation of ERK1/2 MAPK. Similar to our results in Figure 3, alpinumisoflavone treatment with JNK inhibition led to powerful suppression of ERK1/2 signal transduction via interaction, which needs further investigation. This overall trend supports the induction of apoptosis in HCC cells via alpinumisoflavone-induced regulation of the MAPK/PI3K signaling pathway.

Mitochondria are closely involved in cell survival, ATP production, maintenance of calcium homeostasis, and regulation of apoptosis [37]. Furthermore, it has been demonstrated that alpinumisoflavone can induce apoptosis via depolarization of the MMP in human ovarian cancer cells, which is consistent with our results [17]. Maintaining the polarization of the MMP is crucial for mitochondria, and the liver is a major metabolic organ supplying energy to our bodies [38]. Transport of electrons and ATP synthesis occur in the mitochondrial inner membrane via OXPHOS [39]. It has been reported that the inhibition of mitochondrial complex I could activate apoptosis with disruption of the polarization of the MMP in human pancreatic cancer, cervical cancer, and lung adenocarcinoma cells [40,41]. In addition, NUDFA4L2, a component of mitochondrial complex I, was reportedly overexpressed in HCC patients and related to their poor overall survival; thus, this protein was suggested as a new therapeutic target [42]. Similarly, the inhibition of mitochondrial complex III suppressed mitochondrial respiration and induced lung cancer cell death [43]. Moreover, sorafenib is known to induce apoptosis by targeting mitochondrial complexes I and III [44,45]. These previous studies support our hypothesis that apoptosis was activated in response to the inhibition of mitochondrial complexes I and III in alpinumisoflavone-treated HCC cells. Lastly, the different tendency of mitochondrial complex V-related genes and response to respiration between Hep3B and Huh7 cells might be due to the different p53-types present in these two cell lines, as suggested by a colon cancer case [46,47]. p53 protein types are known to affect the sensitivity and resistance of HCC cells to sorafenib, which is mediated by PKCδ [48]. Increasing PKCδ activity via p53 modulation by apigenin, an isoflavone, could overcome chemoresistance in colon cancer [49]. Genistein, another isoflavone-derived extract, shows different sensitivities and anti-cancer effects depending on the p53 type [50]. Thus, drug response and mitochondrial metabolism could change depending on the type of p53 [51], which is consistent with our results regarding responsiveness to OXPHOS and mitochondrial complex-related genes. 

Calcium accumulation in the mitochondrial matrix is a well-known indicator of mitochondria-mediated intrinsic apoptosis. The levels of mitochondrial calcium are controlled by permeable transition pores located in the mitochondrial inner membrane, which is also associated with mitochondrial dysfunction and apoptosis [52]. Alterations in the calcium transport in the mitochondria inhibit HCC cell proliferation [53], and ascorbic acid and sorafenib interfere with mitochondrial depolarization and intracellular calcium homeostasis, leading to HCC death [54]. Furthermore, we confirmed that mitochondrial apoptotic proteins Bcl-xL and BAK were regulated by alpinumisoflavone. Taken together, these results confirmed that alpinumisoflavone induces mitochondrial dysfunction and mitochondria-mediated apoptosis.

## 5. Conclusions

In conclusion, we have demonstrated the anti-cancer effects of alpinumisoflavone and verified its mechanisms in HCC cells. Therefore, we propose alpinumisoflavone as a novel therapeutic agent, which could be combined with sorafenib, for HCC treatment with potentially high efficiency. 

## Figures and Tables

**Table 1:** List of antibodies used in immunoblotting.

Antibody	Catalog No.	Source
p-ERK1/2 (Thr^202^/Tyr^204^)	9101	Cell Signaling Technology
P70S6K (Thr^421^/Ser^424^)	9204	Cell Signaling Technology
p-S6 (Ser^235/236^)	2211	Cell Signaling Technology
p-JNK (Thr^183/^Tyr^185^)	4668	Cell Signaling Technology
p-P38(Thr^180^/Tyr^182^)	4511	Cell Signaling Technology
p-P90RSK(Thr^573^)	9346	Cell Signaling Technology
t-ERK1/2	4695	Cell Signaling Technology
t-P70S6K	9202	Cell Signaling Technology
t-S6	2217	Cell Signaling Technology
t-JNK	9252	Cell Signaling Technology
t-P38	9212	Cell Signaling Technology
BAK	12105	Cell Signaling Technology
Bcl-xL	2764	Cell Signaling Technology
TUBA	Sc-32293	Santa Cruz Biotechnology

**Table 2:** Primer sets used for quantitative RT-PCR analysis.

Gene Symbol	GenBank Accession No.	Forward Primer (5′→3′)	Reverse Primer (3′→5′)
*NDUFS3*	NM_004551.3	CTTTCCTCAGGGATCACACC	GAAGCGCAGAGACAACAGG
*NDUFS4*	NM_001318051.2	TACTGAGGCAGACGTTGTGG	TCTTGAGTCTGGTCCTGTGC
*NUDFS5*	NM_001184979.2	TGAACAGCCCTACAAGATGG	GCCCGAGTATAACCGATTCC
*NDUFS8*	NM_002496.4	CGCTATGACATCGACATGACC	TGTACAGCAGCTCCTCATGG
*MT-ND1*	NC_012920.1	TAGCAGAGACCAACCGAACC	GGCGTATTCGATGTTGAAGC
*MT-ND2*	NC_012920.1	CCGGACAATGAACCATAACC	CTGGGACTCAGAAGTGAAAGG
*MT-ND3*	NC_012920.1	AATCCACCCCTTACGAGTGC	GCTCATGGTAGGGGTAAAAGG
*MT-ND4L*	NC_012920.1	TCGCTCACACCTCATATCC	CGGCAAAGACTAGTATGGC
*MT-ND5*	NC_012920.1	CGCTATCACCACTCTGTTCG	GGAATGCTAGGTGTGGTTGG
*UQCRQ*	NM_014402.5	GGAACCTCTCATCTGCTTCG	AGCTGGATTCTTCCTCTTGG
*UQCRFS1*	NM_006003.3	GCTTCTGCTGATGTGTTGG	CCTGCTCAATTTCCTTCTGG
*MT-CYB*	NC_012920.1	ACTACAACCCTTCGCTGACG	AGAAGAGCGATGGTGAGAGC
*ATP5FIB*	NM_001686.4	TCCTGTTGGTCCTGGACTT	ATGAACTCTGGAGCCTCAGC
*ATP5FIC*	NM_001001973.3	TGCTATTCTTCCTCCATTGC	TGTCACCAATTCCAACAAGC
*ATP5MC3*	NM_001002258.5	TTCGACCAGTGCAATCAGC	CCAATACCAGCACCAGAACC
*ATP5ME*	NM_007100.4	TGTTCCTCGGTGTGGCCTA	CTGCTGCTATCCTCCTCTCC
*ATP5PD*	NM_001003785.2	CTGAGAATCCACCAGCTATCG	TGGCCTTTGAGAGAGACACC
*ATP5PO*	NM_001697.3	ATCCCTATGTGAAGCGTTCC	AACGACTCCTTGGGTATTGC
*GAPDH*	NM_001206359.1	CAATGACCCCTTCATTGACC	ATCACCCCATTTGATGTTGG

## Data Availability

Data are contained within the article.
